# Significant transcriptomic changes are associated with differentiation of bone marrow-derived mesenchymal stem cells into neural progenitor-like cells in the presence of bFGF and EGF

**DOI:** 10.1186/s13578-020-00487-z

**Published:** 2020-10-28

**Authors:** Amir Ali Khan, Tee Jong Huat, Abdullah Al Mutery, Ahmed Taher El-Serafi, Hassen Hadj Kacem, Sallam Hasan Abdallah, Muhammed Faruque Reza, Jafri Malin Abdullah, Hasnan Jaafar

**Affiliations:** 1 Department of Applied Biology, College of Sciences, University of Sharjah, P.O. Box 27272, Emirate of Sharjah, United Arab Emirates; 2 Department of Biological Sciences, Faculty of Science, National University of Singapore, Singapore, 117543 Singapore; 3 Department of Neuroscience, School of Medical Sciences, Universiti Sains Malaysia Health Campus, Jalan Raja Perempuan Zainab II, 16150, Kubang Kerian, Kelantan Malaysia; 4 Department of Biomedical and Clinical Sciences (BKV), Linköping University, P.O. Box 581 83, Linköping, Sweden; 5 Research Institute of Science and Engineering, University of Sharjah, P.O. Box 27272, Emirate of Sharjah, United Arab Emirates; 6 Brain and Behavior Cluster, School of Medical Sciences, Universiti Sains Malaysia Health Campus, Jalan Raja Perempuan Zainab II, 16150, Kubang Kerian, Kelantan Malaysia; 7 Department of Pathology, School of Medical Sciences, Universiti Sains Malaysia Health Campus, Jalan Raja Perempuan Zainab II, 16150, Kubang Kerian, Kelantan Malaysia

**Keywords:** Mesenchymal stem cells, Neural progenitor-like cells, Differentiation, mRNA-seq, EGF, bFGF, Transcriptomic

## Abstract

**Introduction:**

Mesenchymal stem cells (MSCs) isolated from bone marrow have different developmental origins, including neural crest. MSCs can differentiate into neural progenitor-like cells (NPCs) under the influence of bFGF and EGF. NPCs can terminally differentiate into neurons that express beta-III-tubulin and elicit action potential. The main aim of the study was to identify key genetic markers involved in differentiation of MSCs into NPCs through transcriptomic analysis.

**Method:**

Total RNA was isolated from MSCs and MSCs-derived NPCs followed by cDNA library construction for transcriptomic analysis. Sample libraries that passed the quality and quantity assessments were subjected to high throughput mRNA sequencing using NextSeq®500. Differential gene expression analysis was performed using the DESeq2 R package with MSC samples being a reference group. The expression of eight differentially regulated genes was counter validated using real-time PCR.

**Results:**

In total, of the 3,252 differentially regulated genes between MSCs and NPCs with two or more folds, 1,771 were upregulated genes, whereas 1,481 were downregulated in NPCs. Amongst these differential genes, 104 transcription factors were upregulated, and 45 were downregulated in NPCs. Neurogenesis related genes were upregulated in NPCs and the main non-redundant gene ontology (GO) terms enriched in NPCs were the autonomic nervous system, cell surface receptor signalling pathways), extracellular structure organisation, and programmed cell death. The main non-redundant GO terms enriched in MSCs included cytoskeleton organisation cytoskeleton structural constituent, mitotic cell cycle), and the mitotic cell cycle process Gene set enrichment analysis also confirmed cell cycle regulated pathways as well as Biocarta integrin pathway were upregulated in MSCs. Transcription factors enrichment analysis by ChEA3 revealed Foxs1 and HEYL, amongst the top five transcription factors, inhibits and enhances, respectively, the NPCs differentiation of MSCs.

**Conclusions:**

The vast differences in the transcriptomic profiles between NPCs and MSCs revealed a set of markers that can identify the differentiation stage of NPCs as well as provide new targets to enhance MSCs differentiation into NPCs.

## Background

Bone marrow-derived mesenchymal stem cells (MSCs) have a broad differentiation capacity, including their differentiation potential into neural lineages. The differentiation potential may be due to the heterogeneous nature of MSCs [1–3]. During early mammalian development, neural crest cells migrate to the bone marrow and contribute to a subset of MSCs that express nestin, an intermediate filament protein expressed in neural stem cells [4–6]. Human induced pluripotent stem cells (iPSCs) were differentiated into neural crest cells, and the differentiated neural crest cells were further differentiated into nestin-positive MSCs, which confirmed the neural crest cells contribution to MSC populations [7]. Indeed, the iPSCs-derived human MSCs had a comparable differentiation potential to MSCs and could differentiate into adipocytes, chondrocytes, and osteoblasts.

During in vitro culture, MSCs express nestin, which is also expressed by developmental neural stem cells [8]. Nestin expression in MSCs indicates the inherent propensity of MSC differentiation toward neural lineages.

In vivo MSCs were reported to secrete neuroprotective factors in addition to their anti-inflammatory activities and modulation of microenvironment during transplantation [9, 10]. Several studies have also shown that MSCs may also be differentiated into neural lineages in vivo. These studies have demonstrated that MSCs can differentiate into neuron-like cells to be integrated into nervous system [11–15]. Furthermore, these transplantations have effectively improved the neurological function and survival status of animals after spinal cord injuries and other nervous system diseases.

MSCs can be differentiated into neural progenitor-like cells (NPCs) under the influence of bFGF and EGF in suspension (16). MSCs-derived NPCs will be more suitable for cell therapy in neurodegenerative disorders as they are more readily differentiated into neural lineages compared to undifferentiated MSCs [15, 16]. Therapeutically, it may be more effective, however, to transplant endogenous neural stem cells or primary NPCs in neurological disorders but obtaining these endogenous stem or progenitor cells are extremely difficult in addition to ethical restrictions in their extraction. Hence, it is practical to use the MSCs-derived NPCs in neurodegenerative disorders [17, 18]. Therefore, it is will be therapeutically more effective to enhance the differentiation of MSCs into NPCs.

There are generally two approaches to neural differentiation of MSC in vitro. The first approach consists of making NPCs and their subsequent differentiation into neurons and glial cells [19, 20]. The second approach directly differentiates MSCs into neurons and glial cells without prior differentiation of MSCs into NPCs [21, 22]. However, owing to the adherent nature of neurons, the generation of free-floating NPCs will better benefit its future purpose, especially in cell transplantation.

Trans-lineage differentiation is a complex process involving dynamic changes in gene and protein expressions. In our earlier study, we differentiated MSCs into NPCs using growth factor combination, such as epidermal growth factor (EGF) and basic fibroblast growth factor (bFGF) [19]. Other studies, including ours, demonstrated the differentiation of MSCs into NPCs was optimal in term of cell viability and proliferation after three days of induction [19, 23–25]. The derived NPCs can terminally differentiate into glial-like and neuronal-like cells expressing glial-marker and the neuronal marker, respectively.

Nestin and Sox2 are the main NPC markers; these markers though also expressed by MSCs, but their expression is upregulated in the MSC-derived NPCs [26]. In our previous study, we reported that the MSC-derived NPCs expressed Sox2 and Nestin. Giving the more readiness of MSCs-derived NPCs to differentiate into neurons and glial cells compared with MSCs, unravelling differential genes expression between MSCs and MSC-derived NPCs will lead to a better understanding of the molecular processes that govern this differentiation. Knowledge gained from this differential genes expression comparison will lead to a better understanding of the differentiation process which may lead to more effective transplantation strategies for NPCs in neurodegenerative disorders. Previously, we reported the dynamic change in the microRNA profile of MSCs upon NPCs differentiation [27]. We found key microRNAs that are involved in the differentiation of NPCs from MSCs. However, there is still a lack of detailed comparison of transcriptomic profiles of rat MSCs and MSCs-derived NPCs in the rat model. Hence, in this study, we utilised mRNA-Sequencing to uncover the changes in the transcriptomic profile of rat MSCs before and soon after the cells undergo neurogenic differentiation on Day 3. This study sheds further light on the propensity of MSC differentiation toward NPCs and reports that NPCs induction of MSC indeed involves a massive change in transcriptomic profiles.

## Materials and methods

### Primary MSC culture

For this study, Sprague Dawley rats were purchased from the animal facility of Universiti Sains Malaysia. MSCs were extracted from bone marrow tissues of three SD rats using a previously described method [19]. Briefly, three SD rats (4 weeks old) were euthanised using an overdose mixture of ketamine-xylazine (Ilium Troy Laboratory, Blacktown, Australia) via intraperitoneal injection. Femoral and tibial bones were then aseptically dissected, and 5 mL of 20% DMEM was injected into the central canal of the bones to extrude the marrow tissue. Next, the cell mixture was separated using Ficoll-Paque PREMIUM gradient solution (GE Healthcare Bioscience, Uppsala, Sweden), and mononuclear cells were extracted. The collected cells from three SD rats were plated at a density of 1 × 10^6^ marrow cells and incubated in a humidified chamber at 37 °C with 5% CO_2_. After 24 h, floating cells were removed using total media replacement. At 80% confluence, MSCs were detached with TrypLE™ Express stable trypsin replacement enzyme without phenol red (Life Technologies, Carlsbad, CA, USA) and subcultured until passage 3 for neural induction.

### Differentiation of MSCs into NPCs

MSCs at passage 3 (3 biological replicates) were differentiated into NPCs as previously described [19]. Briefly, MSCs (collected from three SD rats) at P3 were plated in three (triplicate) ultra-low attachment plates at a density of 1 × 10^6^ cells/mL and were induced into NPCs with NeuroCult® NS-A proliferation media (STEMCELL Technologies, Vancouver, BC, Canada) supplemented with 20 ng/ml of bFGF (C/N: 4039–10; BioVision, CA, USA), 20 ng/ml of EGF (C/N: PMG8045; Gibco, Life Technologies, Carlsbad, CA, USA) and 1% penicillin–streptomycin (Gibco, Life Technologies, Carlsbad, CA, USA). Cells were monitored daily, and growth factors were supplemented every other day.

### Differentiation of rat MSCs into adipocytes, chondrocytes, and osteocytes

The remaining MSCs after NPCs differentiation and mRNA extraction for mRNA-Seq were grown until passage 4. They were then differentiated into adipocytes, chondrocytes, and osteocytes using the StemPro® Adipogenesis differentiation kit (C/N: A1007001, Gibco, Life Technologies, Carlsbad, CA, USA), StemPro® Chondrogenesis differentiation kit (C/N: A1007101, Gibco, Life Technologies, Carlsbad, CA, USA) and the StemPro® Osteogenesis differentiation kit (C/N: A1007201, Gibco, Life Technologies, Carlsbad, CA, USA), respectively, according to manufacturer protocol. Differentiated adipocytes, chondrocytes, and osteocytes were stained with oil red O, Alcian Blue, and Alizarin Red S solution, respectively, as described previously [28]. Images were visualised under a light microscope and captured using an attached DSLR camera.

### Proliferation analysis

CellTiter 96® AQ_ueous_ One Solution Reagent was used to evaluate NPC viability at 24, 48, 72, and 96 h of culture. Viability rate was determined using the 3-(4,5-dimethylthiazol-2-yl)-5-(3-carboxymethoxyphenyl)-2-(4-sulfophenyl)-2H-tetrazolium (MTS) method. One thousand cells were seeded in 96 well plates and incubated with MTS solution (20 µl, Promega, Milan, Italy) for four hours at 37 °C. The absorbance was read at 490 nm using a spectrophotometer. Non-treated cells (in basal media and without growth factors) were used as control.

### Immunocytochemistry

Cells were fixed with 4% paraformaldehyde for 20 min and permeabilised with 0.012% Triton X-100 in PBS for 30 min. Cells were blocked with 5% BSA/PBS for one hour at room temperature to prevent non-specific binding. The cells were then incubated with primary antibodies overnight at 4 °C and protected from light. The following day, cells were incubated with donkey anti-rabbit IgG Cy3 secondary antibody (EMD Millipore, MA, USA) for one hour at room temperature followed by three washes with chilled 1 × PBS. Nuclei were counterstained with Sytox® blue nucleic acid stain (Life Technologies, Carlsbad, CA, USA) and mounted with fluorescence anti-fade mounting medium (DAKO, Agilent Technologies, CA, USA). Confocal microscopy equipped with Pascal 5 imaging software was used for sample observation.

### Terminal differentiation of NPCs into neuronal-like cells

MSCs-derived NPCs at Day 3 of induction were differentiated into neuronal-like cells in a standardised NeuroCult™ NS-A differentiation media (STEMCELL Technologies, Vancouver, BC, Canada). Briefly, NPCs were incubated with Accutase® cell detachment solution (Merck Millipore, MA, USA) for 5 min at 37 °C and gently triturated into the single-cell suspension. Suspended cells were centrifuged to remove the supernatant, and the cell pellet was then resuspended in the complete NeuroCult™ NS-A differentiation media. Cells were seeded into a poly-d-lysine-coated micro dish at a density of 1 × 10^5^ cells/ml. NPCs were terminally differentiated for 14 days in a humidified chamber at 37 °C with 5% CO_2_ supply.

The functional properties of neuronal-like cells differentiated from NPCs were assessed using a whole-cell patch-clamp recording. Briefly, neuronal-like cells were continuously superfused in artificial cerebrospinal fluid at room temperature throughout the recording. Microelectrode pipettes with an input resistance of 5 to 8 MΩ were fabricated from borosilicate glass capillary tubing using a P-97 micropipette puller. The recording microelectrode pipette was then filled with an intracellular solution. The microelectrode was placed in the pipette holder, and positive pressure was applied. Target cells were slowly approached until changes in the test pulse amplitude were observed. Once a steady resistance was obtained, the positive pressure was released rapidly, and the resistance gradually increased until a giga-ohm (GΩ) seal was formed. Continuous suction was then applied until the membrane broke as evidenced by a change in the capacitance and the test pulse current. The miniature postsynaptic current was recorded using a pClamp program interface. The recording was performed in triplicate, and a minimum of three cells from each group was recorded.

### Total RNAs extraction and illumina cDNA library preparation

At passage 3, mRNAs were extracted from the MSCs from three rats (three biological replicates), and NPCs differentiated from the three biological replicas of MSCs on Day 3. Total RNAs were extracted from the six samples using a miRNeasy mini kit (Qiagen, Hilden, Germany) according to the manufacturer’s protocol. The cDNA library construction was performed using the TruSeq® Stranded mRNA sample preparation kit (Illumina, CA, USA). Briefly, 300 ng of total RNA from each sample was enriched for poly-A containing mRNA using poly-T oligo-attached magnetic beads followed by mRNAs fragmentation at 94 °C for 8 min and held at 4 °C. The fragmented mRNAs were subjected to first-strand cDNA synthesis using Super-Script II reverse transcriptase (Invitrogen, Carlsbad, CA). The cDNAs were further converted into double-stranded cDNA and were purified using Agencourt AMPURE XP system (Beckman Coulter, CA, USA) to eliminate the reaction mix.

Next, the fragments were adenylated at 3′ ends and ligated onto the complimentary Illumina sequencing adaptors, and the mRNA stranded libraries were synthesised. After a clean-up step, the libraries were then amplified with PCR followed by streptavidin magnetic bead purification and two-step hybridisation reactions to obtain a pool of different indexing libraries. Finally, the quality and quantity of the sequencing library were assessed using a Bioanalyzer DNA high sensitivity chip (Agilent Technologies, CA, USA) and quantitative real-time PCR, respectively. High throughput next-generation sequencing was performed using Nextseq®500 Sequencing System (Illumina, CA, USA) according to the manufacturer’s instruction, by setting a single-end sequence of 50 M reads per sample.

### Read pre-processing, QC, alignment and gene quantification

Raw reads were evaluated for quality checks in terms of sequencing quality and contamination. Reads were trimmed and filtered using BBDuk [29]. Reads were then aligned to the latest reference genome (rn6) with GTF from Ensembl (v99) using STAR aligner [30]. Aligned reads were transformed into read counts per gene using the RSEM tool [31].

### Differential expression analysis

Pairwise differential expression analysis was performed using the DESeq2 R package with MSC samples being a reference for individual experiments [32]. Briefly, expression counts were scaled and normalised to correct the sequencing depth and batch differences among samples for the pairwise group. These normalised counts were then used for differential expression analysis and to generate fold change values in log2 scale [log2(sample/control)] to contrast NPCs against MSCs. Genes with lower read count can generate higher fold change values, which may lead to possible false positives. Hence, to adjust the fold changes that arise due to the ratio between lower read counts in samples, we employed the fold-change shrinkage estimator approach from DESeq2. Genes with an adjusted P-value ≤ 0.05 and log2fold change of + 1 or − 1 were considered as significantly up- and downregulated genes.

### Gene ontology enrichment analysis

Gene ontology analysis was performed on up- and downregulated genes from different analysis with the Cytoscape v3.6.1 with ClueGO v2.5.5 plugin [33]. Statistically enriched biological processes (Bonferroni step down adjusted p-value <  = 0.001) (updated on 20/05/2019). In order to reduce the redundancy, the GO terms were functionally grouped according to their k-score, and the most significant GO term of each group was used to summarise the GO enrichment analysis result. The full list of significantly enriched GO terms are reported in Additional file 3 and the representative GO terms are shown in Fig. 4.

### Gene set enrichment analysis

Gene set enrichment analysis (FDR < 0.25) was performed as described previously against the genesets from MSigDB with the ranking metric signal-to-noise with 1,000 geneset permutations for statistical assessment of enrichment [34]. The rat genes were ‘humanised’ by converting the rat gene expression profiles using Ensembl Biomart before performing GSEA [35].

### Pathway analysis

Pathway analysis was performed using the Reactome analysis tool for up- and downregulated genes [36]. Pathways with FDR ≤ 0.05 were considered significant.

### Transcription factor analysis

The reported functioning of genes as transcription factors was downloaded from the TFcheckpoint database [37], and reported Entrez gene IDs were converted to corresponding Ensembl ids using Ensembl Biomart. ChEA3 database web-server application was used on the differentially expressed genes, to perform transcription factor (TF) enrichment analysis. Briefly, 3,253 differentially expressed rat genes were humanized and gene symbols with human orthology confidence of 1 (high) were used as input in ChEA3 [38].

### Quantitative real-time PCR validation

The differential expression of the eight genes was validated using quantitative RT-qPCR. RT^2^ Profiler PCR Arrays were used to assess the quantitative expressions of *Mmp10*, *Gria2*, *Frzb*, *Cass4*, Kif2c, *Casq2,* and Tagln (Additional file 4). The PCR array analyses were performed by Exiqon. A/S (Vedbaek, Denmark). The catalogue number for each gene is provided in Additional file 4. RNA samples were converted into first-strand cDNAs using QIAGEN RT^2^ First Strand Kit (Qiagen, Venlo, Netherlands). The first strands were used as the templates for the PCR. Then, the cDNA templates were mixed with RT^2^ qPCR master mixes and aliquoted into each well of the same plate containing pre-dispensed gene-specific primer sets. Relative gene expressions were calculated using the ddCt method, and the fold changes are listed in the excel sheet as (2^-(Ct (GOI)-mean Ct (HKG)) for all biological and technical replicates (M = MSCs and A = NPCs). The mean of the gene fold changes was calculated from the three biological and technical replicates. The bar charts (Additional file 4) represents relative gene expression of genes with error bars between MSCs and NPCs. The housekeeping genes used in the study were *B2m*, *Hprt1* and, *Rplp1.* Three RNA and PCR quality controls were also used in the array.

## Results

### Characterisation of rat MSCs

**Fig. 1:**
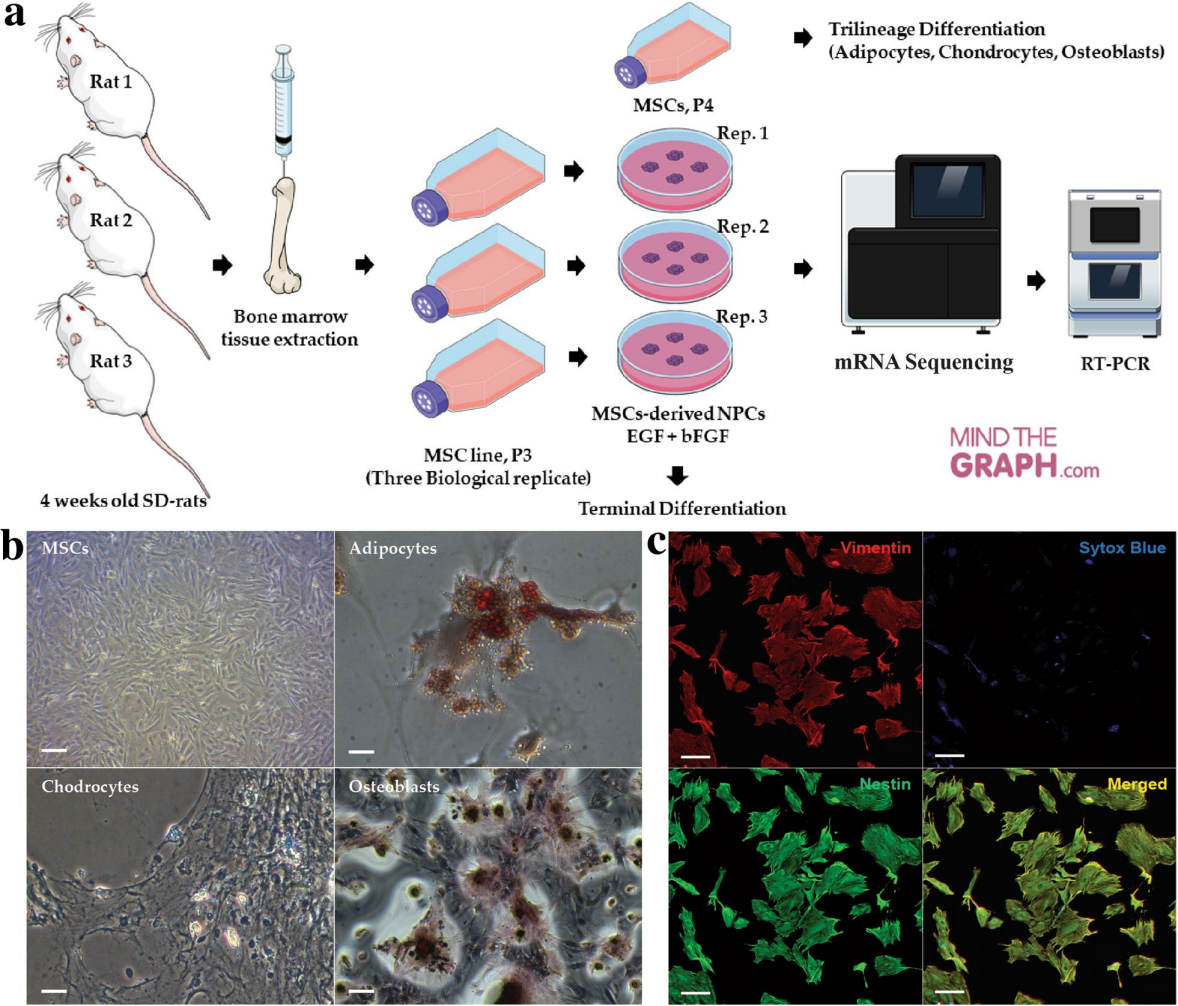
Study design and primary culture of rat MSCs. **a** An overview of study design. **b** Rat MSCs at passage four and trilineage differentiation of MSCs into adipocytes, chondrocytes, and osteoblast cells. Differentiated adipocytes, chondrocytes, and osteocytes were stained with oil red O, Alcian Blue and Alizarin Red S solution respectively and images were viewed under an inverted light microscope at 10 × magnification; scale bar = 50 µm. **c** Representative figure of MSCs expressing vimentin and nestin. Co-expression of vimentin and nestin confirms the true expression of nestin and vimentin in rat MSCs (Merged). Images were viewed under the confocal microscope at 10× magnification, scale bar = 100 µm

An overview of the experimental design is outlined in Fig. 1a. MSCs were extracted from the bone marrow of three Sprague–Dawley (SD) rats (three biological replicates) and were expanded in vitro from passage 0 to passage 2. MSCs were cryopreserved at passage 2, and subsequent experiments were conducted using the MSC line at passage 3. Leftover MSCs after NPCs differentiation and extraction of total mRNAs were differentiated into adipocytes, osteoblast, and chondrocytes. The MSCs were viable and capable of trilineage differentiation into adipocytes, chondrocytes, and osteocytes (Fig. 1b). Phenotypic analysis through immunocytochemical staining indicated that MSC expressed nestin, which co-localised with vimentin (Fig. 1c). Cytofluorimetric evaluation that we reported in our previous study confirmed that MSCs were positive for CD90 (86.8%), CD44 (35.4%), fibronectin (98.3%), vimentin (90.9%), and nestin (85.6%). However, they were negative for the macrophage marker, CD11b (2.0%) [19].

### Differentiation of MSCs into NPCs

**Fig. 2:**
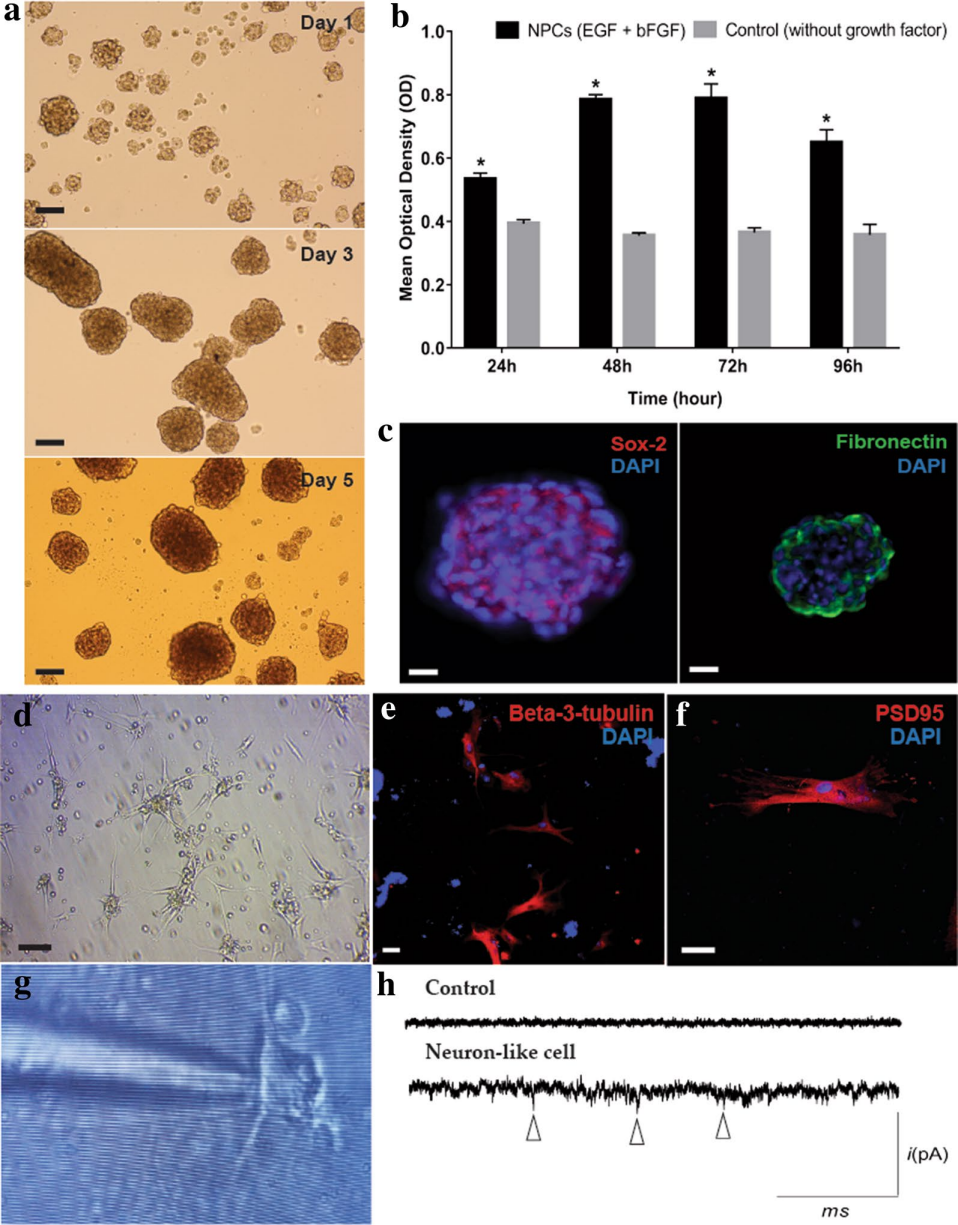
Characterisation and terminal differentiation of MSCs-derived NPCs. **a** Rat MSCs detached from the culture surface and formed free-floating neurospheres (Day 1, Day 3, and Day 5). Images were viewed and taken under an inverted light microscope at 20 × magnification; scale bar = 50 µm. **b** Cell viability of NPCs determined using MTS assay at days 1, 2, 3, and 4 post-induction. The mean ± SD of three independent experiments are shown. *P < 0.05. **c** NPCs positively expressed marker used to characterise neural stem cell, Sox-2, and showed low expression of fibronectin. Images were taken under confocal microscopy at 20 × magnification; scale bare = 40 µm. **d** NPCs adhered to the culture surface and differentiated into cells with neuronal-like morphology. The representative image was taken under an inverted light microscope at 20 × magnification, scale bar = 50 µm. **e** Beta-3-tubulin and **f** PSD-95 expression indicating the neurons. Images were taken under confocal microscope at 20 × (scale bar = 20 µm) and 40 × (scale bar = 50 µm), respectively. **g** Microelectrode pipette approaches a neuronal-like cell during whole-cell patch-clamp recording. The image was taken using a phone camera. **h** Representative figure of miniature excitatory postsynaptic current (mEPSC) of neuronal-like cells showing spikes (∆) compared to undifferentiated rat MSCs. Cells were recorded in voltage-clamp at -60 mV

The MSCs were differentiated into NPCs as described in the method. Free-floating sphere-like cells were observed in the presence of EGF and bFGF after 24 h of differentiation (Fig. 2a). Cell proliferation assay showed that the sphere-like cells were viable (Fig. 2b) with cell growth highest at Day 3, and a noticeable dark core at the centre of the spheres was observable on Day 5. Another study also reported the presence of these dark cores in neural spheres [39]. Therefore, to avoid the effect of apoptotic cells in the centre core of the neurospheres, NPCs on Day 3 were used for subsequent experiments. We reported the characterization of MSC-derived NPCs in one of our earlier studies [19]; in the study, we reported 53.4% of Nestin expression in MSCs suspended in basal media. The flow cytometry analysis showed that Nestin was increased to 94.0% in the MSC-derived NPCs under the influence of EGF and bFGF. Moreover, gene expression for Nestin and Neurofilament (NEFL) was also assessed by real time PCR [19]. The expression of the two genes was significantly increased in NPCs compared to the control.

In the current study we also measured, the expression of Sox2 in NPCs, which is the marker for neural progenitor stem cells. Indeed, expression of Sox2 was increased in MSC-derived NPCs (Basal media + EGF + bFGF) (47.6%) compared to the control Group E (MSCs in basal media only) (18.5%), (Additional file 1: Figure S1). The expression of the Sox2 was also confirmed by Immunocytochemistry (Fig. 2c).

The enhanced expression of these specific neuronal markers in the neurospheres indicated the differentiation of MSCs into NPCs. Fibronectin was also expressed in NPCs (93.1%) (Fig. 2c).

### Terminal differentiation of NPCs

MSCs-derived NPCs differentiated into neuronal cells upon the removal of EGF and bFGF. NPCs adhered to the poly-D-lysine coated surface and differentiated into cells with elongated processes after 14 days in culture (Fig. 2d). Terminally differentiated cells expressed the neuronal marker beta-3-tubulin (Fig. 2e) and postsynaptic marker, PSD95 (Fig. 2f). Moreover, the original current traces of mEPSC recorded in an individual neuronal-like cell showed increasing amplitude compared to current traces recorded in MSCs (control) (Fig. 2g, h). Approximately 50% of NPCs were differentiated into neurons and expressed beta-tubulin III (Additional file 1: Figure S2).

### Transcriptome analysis of NPCs at the day 3 of induction and MSCs

The whole transcriptome of MSCs and MSCs-derived NPCs was investigated using mRNA-seq. The transcriptomic analysis was conducted using total RNAs isolated from the three biological replicates of MSCs and MSCs-derived NPCs, a total of six samples (Fig. 1A). Quality control and mapping statistics showed that 96% of the reads (corresponding to 51.86 M reads) passed the filtering criteria across all samples (Additional file 2). Using the STAR alignment tool, approximately 97% of the reads corresponding to 50.5 M reads mapped on the genome across all samples. Adapter sequences were trimmed, and high-quality reads were mapped to the rat reference genome.

**Fig. 3:**
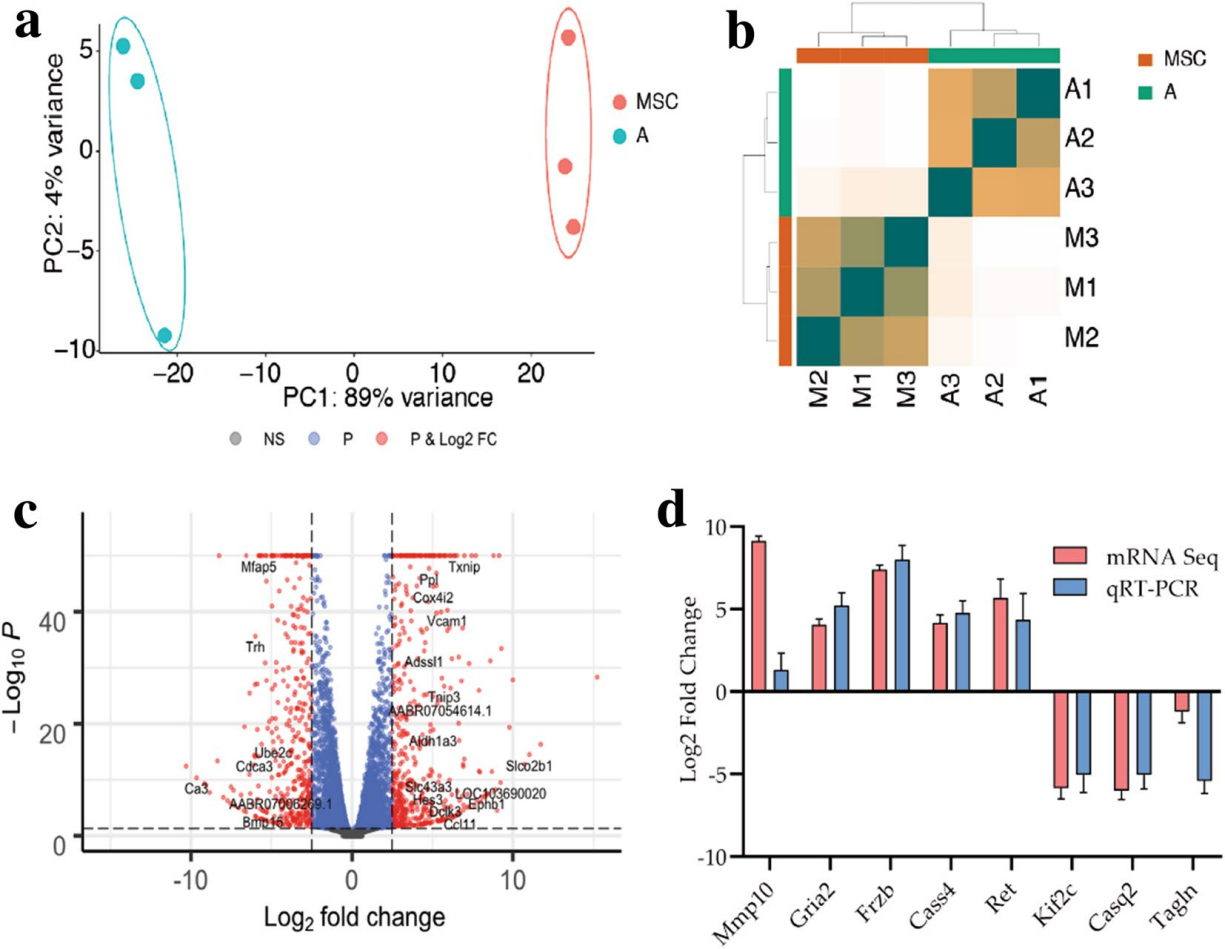
**a** PCA plot of samples: Samples are colour coded based on their group. PCA method reduces the spatial dimensions of the gene expression pattern among samples and cluster the datasets based on their similarity. Results above show that the MSC samples are well separated from NPCs. **b** Unsupervised hierarchical sample clustering. Samples were hierarchically clustered using the sample distance calculated from the overall dispersion rate of gene expression. Results above show that the MSC samples cluster together and are separated from NPCs. **c** Volcano Plot: Illustration of differentially expressed genes for each comparison of NPCs versus MSC samples. For illustration purposes, a cut-off of 0.05 and ( ±) 2.5 is used for P-value and fold change respectively to highlight the most significant differentially expressed genes (highlighted in red). Significantly differentially expressed genes are annotated. P-values are capped at 50 for maximised view. **d** Fold changes of the respective genes in NPCs relative to rat MSCs; comparing the expression obtained from mRNA-Seq and RT-qPCR. Values of the RT-qPCR were normalized with the average values of the housekeeping genes

Before the differential gene expression analysis, normalisation was performed for read counts differences due to sequence depth and gene lengths variations. Transcripts per kilobase of transcript per million mapped reads were applied to normalise the number of reads for genes to the total number of mapped reads. The overall similarity between the replicates using principal component analysis (PCA) (Fig. 3a) and unsupervised hierarchical clustering using the distance matrix (Fig. 3b) was performed. Overall, the PCA analysis revealed that the two groups—A (MSCs-derived NPCs) and B (MSCs)—were well separated and that the samples clustered together in a manner that corresponded to the group and its corresponding replicates are clustered together The differentially expressed genes with two-fold and above were analysed between NPCs and MSCs.

A total of 3,252 genes were differentially expressed with two or more folds. Additional file 3 contains all of the differentially regulated genes. Figure 3c shows the volcano plot of the analysis, which demonstrates a significant magnitude of change in the expression values of some representative genes. The plot reveals a considerable number of differentially regulated genes.

Out of 3,252 differentially regulated genes, 1,771 were upregulated, whereas 1,481 were downregulated in NPCs. The top 20 significantly differentially expressed genes are listed in Table 1. All the genes with their TPM values and differentially regulated genes with their fold changes are listed in Additional file 3.

**Table 1:** Summary statistics of the 20 most differentially regulated genes between NPCs and MSCs

Ensembl	Gene type	Genes symbol	Log2 Foldchange	Adjusted P-value
ENSRNOG00000021201	protein_coding	Txnip	6.97	2.2E−54
ENSRNOG00000014333	protein_coding	Vcam1	5.92	5.3E−41
ENSRNOG00000008245	protein_coding	AABR07054614.1	5.48	5.3E−25
ENSRNOG00000052070	protein_coding	Aldh1a3	5.04	1.2E−19
ENSRNOG00000007827	protein_coding	Cox4i2	5.04	3.0E−45
ENSRNOG00000016456	protein_coding	Il33	− 5.39	3.6E−200
ENSRNOG00000017976	protein_coding	Slco2b1	11.02	3.1E−15
ENSRNOG00000015529	protein_coding	Cdca3	− 6.05	4.1E−15
ENSRNOG00000010079	protein_coding	Ca3	− 9.65	4.4E−11
ENSRNOG00000005906	protein_coding	LOC103690020	9.24	3.2E−10
ENSRNOG00000021410	protein_coding	Negr1	− 9.16	4.3E−10
ENSRNOG00000021260	protein_coding	Prnd	− 9.07	9.2E−10
ENSRNOG00000007865	protein_coding	Ephb1	8.38	2.7E−08
ENSRNOG00000056151	protein_coding	AABR07007642.1	8.31	3.5E−08
ENSRNOG00000011989	protein_coding	Vat1l	6.67	4.9E−07
ENSRNOG00000033026	protein_coding	Dclk3	5.80	5.2E−07
ENSRNOG00000014751	protein_coding	Ret	5.68	2.5E−06
ENSRNOG00000029342	protein_coding	Scn7a	5.68	6.2E−06
ENSRNOG00000036674	protein_coding	Cd7	7.14	8.2E−06
ENSRNOG00000009629	protein_coding	Car2	5.88	8.9E−06

### Dataset validation by quantitative real-time PCR (RT-qPCR)

Eight genes with various differential folds were chosen from the gene list in Table 1 and Additional file 3 for dataset validation using RT-qPCR. The expression of five upregulated genes (*Mmp10*, *Gria2*, *Frzb*, *Cass4,* and *Ret)* and three downregulated genes (*Kif2c*, *Tagln,* and *Casq2*) were quantified by RT-qPCR (Fig. 3d). The RT-qPCR confirmed the analogous differential regulation of these genes in NPCs to that of the mRNA sequence dataset (Additional file 4). Hence, it validates the gene expression patterns in the sequencing data.

### Gene ontology term and pathway enrichment analysis

**Fig. 4:**
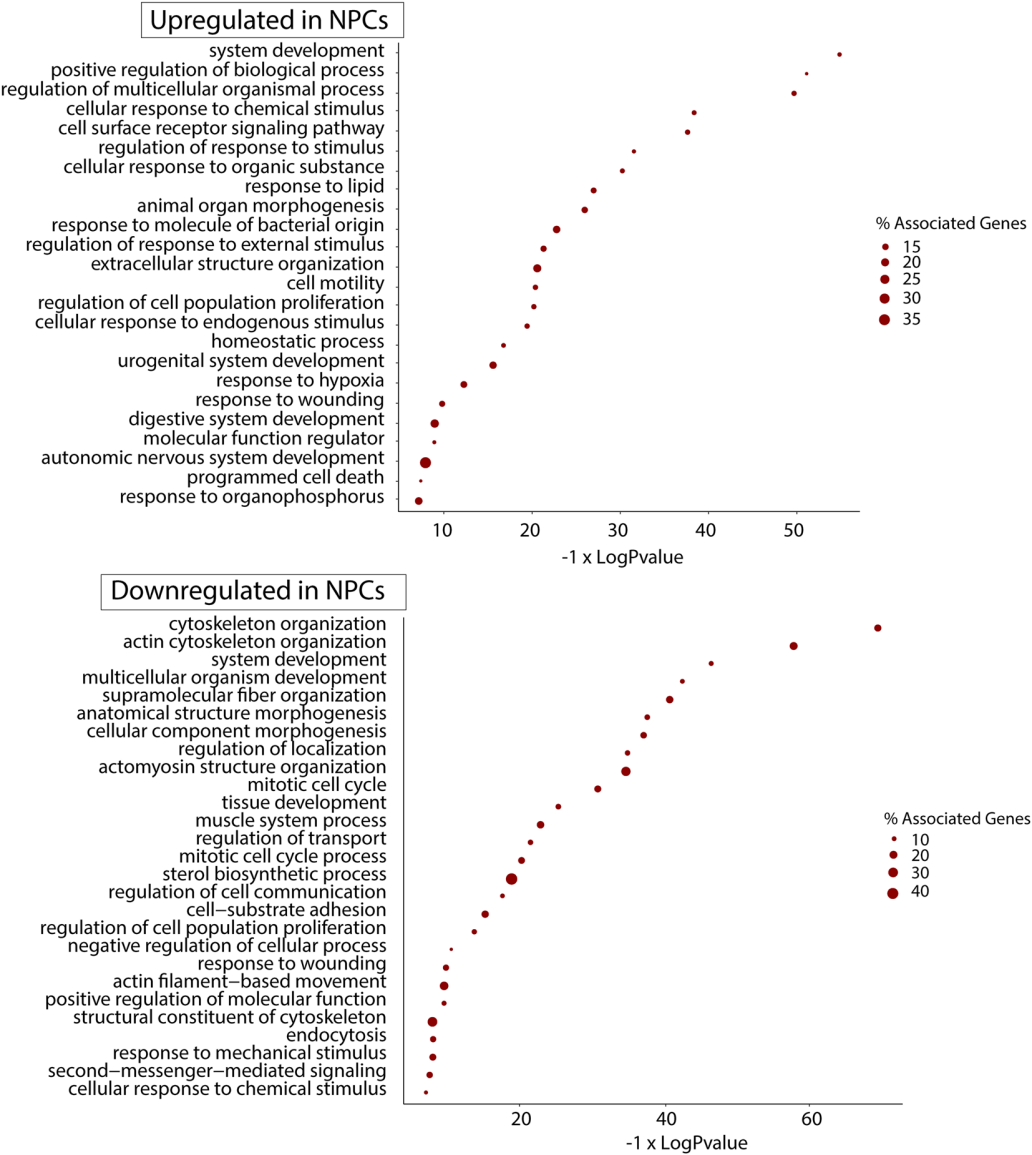
Gene ontology terms enriched in upregulated and downregulated genes in MSCs-derived NPCs. Significantly enriched (right-sided hypergeometric test) GO terms are shown in the y-axis, and the corresponding adjusted P-value (Bonferroni step down) are reported in the x-axis. The size of the dots reports the percentage of genes associated with the GO term identified in the upregulated genes

All the differentially regulated genes were selected for gene ontology (GO) enrichment annotation and were analysed in ClueGO v2.5.5. Non-redundant GO terms (after semantics similarity) that were significantly over-represented in the list of up- and downregulated genes in NPCs were obtained (Fig. 4). Figure 4 lists the main GO terms that were enriched in the differentially regulated genes. The full list of all of GO terms is listed in Additional file 3. The main non-redundant GO terms enriched in NPCs and were related to neurogenesis and stem cells differentiation included autonomic nervous system, cell surface receptor signalling pathways, extracellular structure organisation, and programmed cell death (Fig. 4). The differentiation of MSCs into NPCs may involve many changes in other molecular processes. Consequently, other GO terms were also annotated during the differentiation. We only focused on the GO terms related to neurogenesis, signalling pathways, extracellular matrix and programmed cell death as these processes are crucial for MSCs differentiation into NPCs. While the relevant GO terms that were enriched in MSCs such as cytoskeleton organisation, structural cytoskeleton constituent and mitotic cell cycle process were related to the undifferentiated growth and morphological characterises of the in vitro growth of MSCs.

### Gene set enrichment analysis (GSEA)

**Fig. 5:**
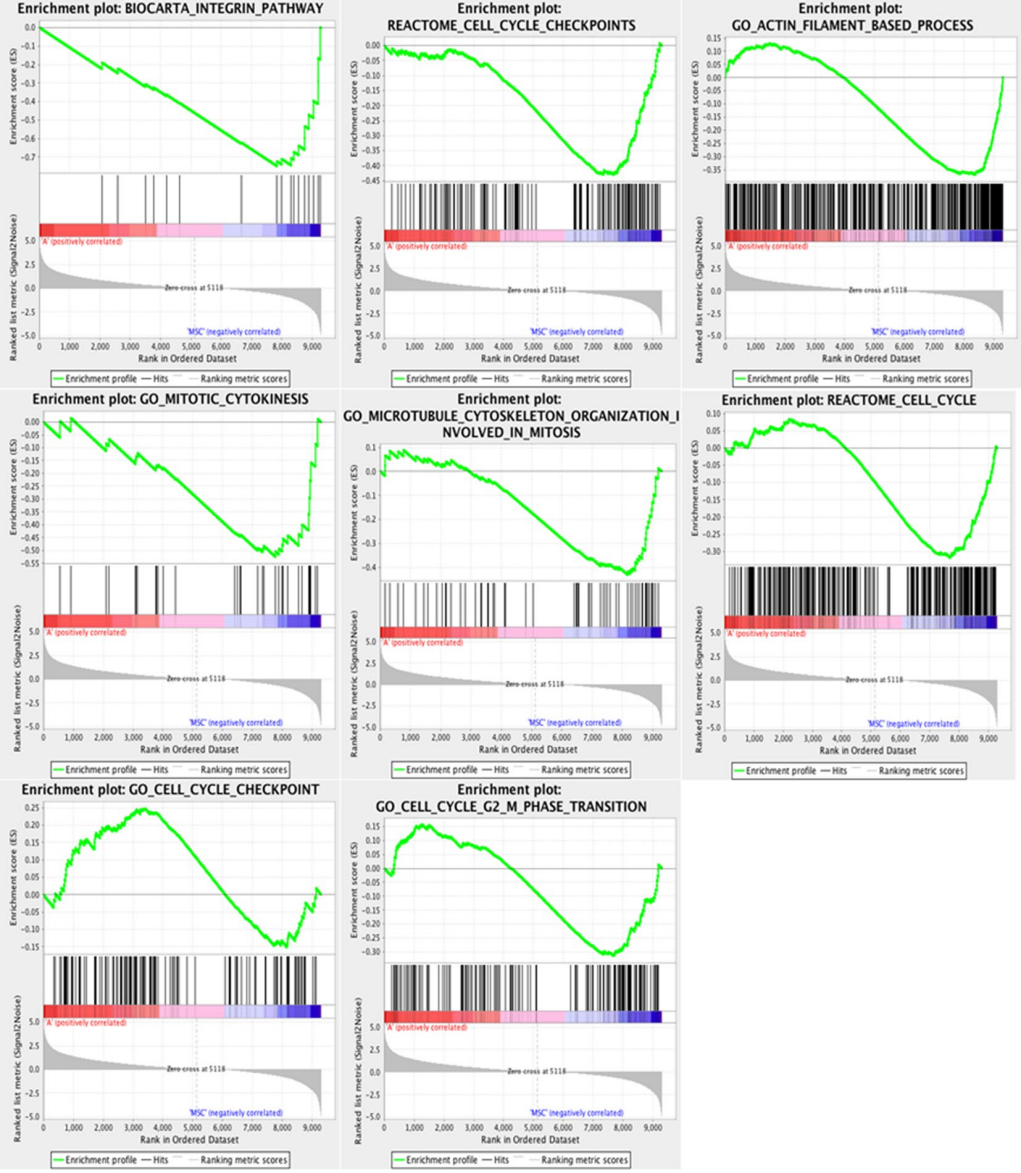
Gene set enrichment. Gene sets from MSigDB significantly enriched are reported for gene expression analysis NPCs vs MSCs. All the terms reported are upregulated in MSCs

All the differential regulated genes were ranked, and then GSEA was performed. GSEA detects even slight coordinated changes in gene sets and identifies pathways not possible with other analysis. GSEA analysis identified eight pathways or biological process from MSigDB that were upregulated in MSCs (Table 2 and Fig. 5). GSEA plots suggested that the integrin pathway, cytoskeletal organisation, and the cell-cycle related process were upregulated in MSCs. Hence, these processes may have roles in MSC differentiation towards NPCs.

**Table 2:** List of pathways and biological process

MSigDB Gene Set	Number	ES	NES	FDR q-value
BIOCARTA_INTEGRIN_PATHWAY	19	− 0.748	− 1.978	0.004
REACTOME_CELL_CYCLE_CHECKPOINTS	160	− 0.432	− 1.698	0.039
GO_ACTIN_FILAMENT_BASED_PROCESS	435	− 0.370	− 1.613	0.048
GO_MITOTIC_CYTOKINESIS	32	− 0.525	− 1.567	0.056
GO_MICROTUBULE_CYTOSKELETON_ORGANISATION_INVOLVED_IN_MITOSIS	66	− 0.430	− 1.512	0.064
REACTOME_CELL_CYCLE	343	− 0.318	− 1.383	0.121
KEGG_CELL_CYCLE	80	− 0.357	− 1.285	0.193
GO_CELL_CYCLE_G2_M_PHASE_TRANSITION	162	− 0.315	− 1.244	0.193

Reported are the terms significantly enriched (FDR < 0.25) in MSCs. *ES* enrichment score, *NES* normalised enrichment score

### Transcription factor analysis

**Fig. 6:**
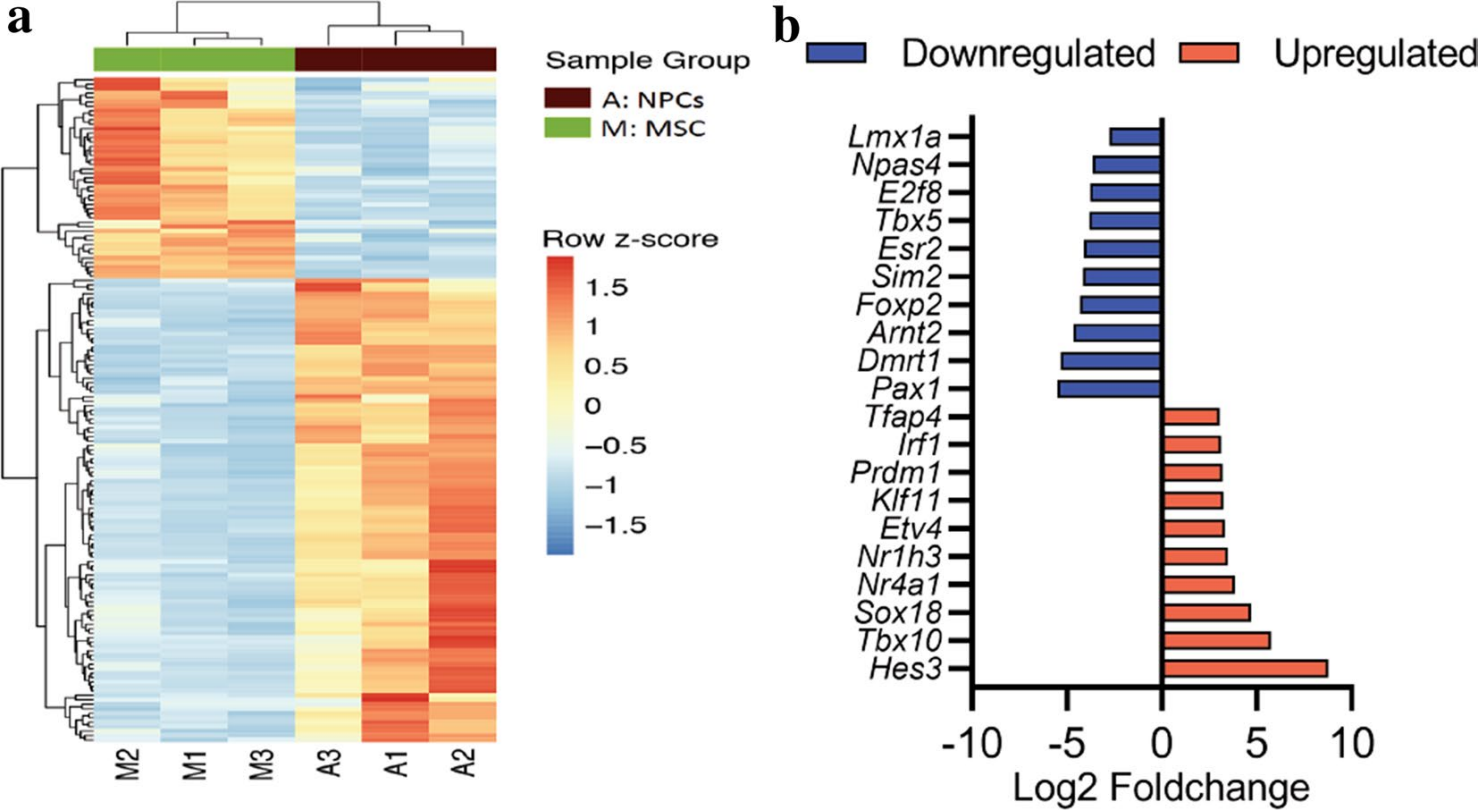
Transcription factor analysis. **a** Hierarchical clustering of differentially expressed transcription factors. Data shown are the significantly differentially expressed transcription factors. The colour scale bar shows z-score values after z-score row normalisation. The heatmap was generated using the heatmap package from R. **b** Top 10 upregulated and downregulated transcription factors with their average fold changes in NPCs

**Fig. 7:**
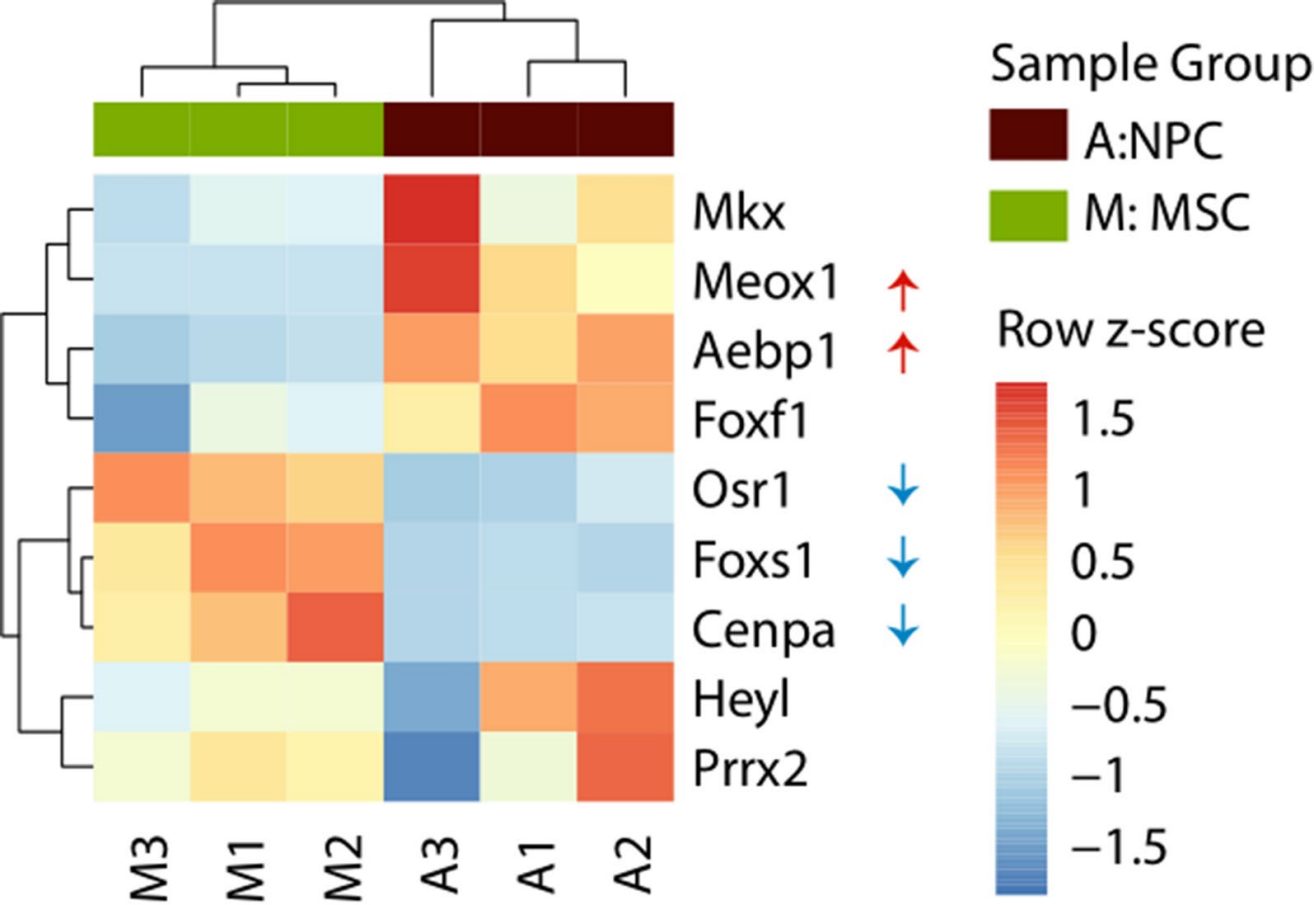
Shown are the top 10 ranked enriched transcription factor from ChEA3 analysis with significantly upregulated (red arrows) and downregulated (blue arrows) TFs in NPC. Gene Tcf21 is not shown as it has zero values across all the samples. The colour scale bar shows TPM values after z-score row normalization. Heatmap was generated using pheatmap package from R

Specific transcription factors have crucial roles in stem cell differentiation and linage commitment. All the differential regulated genes were mapped with the transcription factor checkpoint database. A total of 149 transcription factors were differentially expressed between NPCs and MSCs; 104 transcription factors were upregulated in NPCs, whereas 45 were downregulated. Unsupervised clustering of transcription factors (Fig. 6a) demonstrated that samples within the group were consistent in expression and that samples clustered according to the group; Fig. 6b also lists top ten upregulated and downregulated transcription factors during the differentiation. Additional file 5 contain all the differential regulated transcription factors reported in the differentiation. To gain insight into the specific transcription factors that may be associated to the observed gene expression changes and potentially play roles in the NPC differentiation, the list of 3252 differentially expressed genes were subjected to transcription factor enrichment analysis using ChEA3 [38] (Additional file 3). Figure 7 shows the top 10 ranked enriched transcription factors from ChEA3 analysis with significantly upregulated (red arrows) and downregulated (blue arrows) TFs in NPCs.

## Discussion

The transcriptomic analysis is an essential tool for understanding the molecular processes during stem cells differentiation. In our current and previous studies, we successfully extracted MSCs from the bone marrow of SD rats, which expressed CD90, nestin, fibronectin, and vimentin [19]. Once the successful platform of MSCs was established, they were then differentiated into NPCs in the presence of bFGF and EGF. NPCs attained optimum viability and optimum proliferation on Day 3. Other studies have also reported MSCs differentiation into NPCs after three days of induction in the presence of bFGF and EGF [23–25].

In the previous study, we differentiated NPCs into neuronal-like and glial-like cells [19]. In this study, we assessed the derived neuron functionality through action potential that can be elicited. Hence, the derived neurons were able to elicit the action potential. Furthermore, the neurons expressed the beta-tubulin III, a marker of mature neurons, and they also expressed the postsynaptic density protein 95 (PSD-95), indicating that the derived neurons communicate with each another [40]. Hence, NPCs can be formed on Day 3 of induction from MSCs in the neural media containing bFGF and EGF, and the derived NPCs can be differentiated into glial and neuron cells. These analyses confirmed that MSCs were differentiated into NPCs.

The main aim of this study was to compare the transcriptomes of MSCs and MSC-derived NPCs (on Day 3 of induction) to unravel the molecular processes involved in early neural differentiation of MSCs.. Sequencing was conducted using the Nextseq®500 sequencing system by setting a single-end sequence of 50 million reads per sample. The details of quality control and mapping are provided in Additional file 2. Briefly, 96% of the reads corresponding to 51.86 million reads pass the filtering criteria across all samples, and approximately 97% of the reads corresponding to 50.5 million reads mapped on the genome across all samples.

The global transcriptomic analysis revealed 3252 differentially regulated genes with two or more folds between MSCs and NPCs; 1771 of them were upregulated, whereas 1481 were downregulated in NPCs. The profound changes in the transcriptomes during the differentiation indicate that NPCs have different expression profiles indicating the MSCs were differentiated into NPCs. Eight of the differentially regulated genes were analysed using RT-qPCR, and their differential regulations were analogous to that reported in mRNA sequencing (Additional file 5), providing confidence regarding the other differential expressed genes in mRNA sequencing.

All the differentially expressed genes were assessed for GO term enrichment. All the details of the GO terms, including the number of associated genes, level of GO terms, and statistical significance values, are provided in Additional file 3. Figure 4 lists the main non-redundant GO terms that were enriched either in MSCs or NPCs. The main GO terms related to neural and stem cells differentiation enriched in NPCs included the autonomic nervous system, cell surface receptor signalling pathways, extracellular structure organisation, and programmed cell death.

Fifteen genes from the autonomic nervous system GO term (GO:0048483) were upregulated in NPCs (Additional file 3): *Ednra, Ednrb, Egr2, Fn1, Gbx2, Hes3, Kif26a, Ntrk1, Plxna3, Ret, Sema3a, Sema3f, Six1, Sox8, and Vcam1. Ret* was also confirmed by RT-qPCR. *Ret* signalling has a crucial role in the development of the peripheral and central nervous system and the enteric gut system [41–43]. The upregulation of these neural related genes suggested that the NPCs were neural progenitor-like cells.

Results revealed that 291 genes involved in the cell surface receptor signalling pathways GO term (GO:0007166) were upregulated in NPCs. This GO term lists the signalling pathways that are activated by the binding of the ligand and activate the transcription factors. One of the genes Frzb, which was also validated by RT-qPCR, is a component of the Wnt pathway, which plays a role in the proliferation of neural stem cells, neuronal differentiation, and development [44, 45].

Another GO term related to NPCs differentiation was programmed cell death (GO:0012501). Our previous study showed that there was some apoptosis during the differentiation of MSCs into NPCs. We enhanced the differentiation by adding IGF-1, which in turn enhanced cell proliferation and inhibited apoptosis [19]. In this transcriptomic analysis, 186 genes related to programmed cell death GO term were upregulated in NPCs.

Hence, it indicates that programmed cell death was occurring in some cells when MSCs was differentiating into NPCs.

Sixty-one genes of the extracellular structure organisation GO term (GO:0043062) were upregulated in the NPCs. A microarray study revealed that the extracellular matrix (ECM) genes were upregulated in the NPCs derived from human MSCs [46]. In our study, ECM proteins, such as *Mmp10*, *Mmp3, Mmp13*, *Timp4, Sparcl1, and fibronectin (Fn1*) as well as various types of collagen family genes (*Col10a1, Col15a1, Col16a1, Col17a1, Col18a1, Col23a1, Col25a1, Col27a1, Col3a1, Col4a4, Col5a3, and Col6a1*) were upregulated in NPCs (Additional file 3). This data reflects the roles of extracellular modelling in the differentiation of MSCs into NPCs. Matrix metalloproteinase-10 (*Mmp10*) expression was also confirmed with RT-qPCR. Recent research indicates that *Mmp1*0 has a role in promoting neurogenesis and neuroprotection in the human brain [47]. Hence, it indicates the differentiation of MSCs into NPCs involves extensive extra cellular remodelling.

The main relevant GO terms enriched in MSCs or downregulated in NPCs were: cytoskeleton organisation (GO:00070109), actin cytoskeleton organisation (GO:0030036), structural cytoskeleton constituent (GO:0005200), mitotic cell cycle (GO:0000278), and mitotic cell cycle process (GO:1903047). These results were expected as MSCs possessed cytoskeletal organisation to maintain the fibroblast-like morphology in cell culture. The cytoskeletal organisation is also involved in the differentiation of MSCs. One study reported that mechanical stretches are responsible for cytoskeletal organisation enhanced the differentiation of MSCs into osteoblasts [48]. Another study reported that the organisation of actin modulated MSC migration [49].

Interestingly, Peng et al. reported that cytoskeletal organisation was a crucial step during the differentiation of MSCs into neural lineages [50]. The authors applied RhoA kinase inhibitor, which enhanced the differentiation of MSCs into neural lineages. The derived cells expressed more nestin and MAP2 compared to the control. Another study also revealed that cytoskeletal rearrangement was a critical component during neural development [51]. The cytoskeleton changes take place with signalling pathways and environmental cues. These polymers are formed due to non-covalent bonds which make it easy to form or dismantle. The differentiation of MSCs involves changes in morphology that require cytoskeletal reorganisation [52].

During gene set enrichment analysis (GSEA) differential genes were ranked based on their fold changes and statistically significancant. After ranking, GSEA was performed on certain gene sets from MSigDB to calculate their enrichment. GSEA revealed that the integrin pathway and cytoskeletal organisation were enriched in MSCs. One study reported that integrin enhanced the differentiation of MSCs into osteoblast [53]. Other studies also confirmed the roles of integrin pathway in MSC differentiation [54, 55]. Furthermore, integrin interacts with cytoskeletal proteins to mediate their effects on cells [56, 57]. In this way, integrin can mediate the interaction between cytoskeletal proteins and ECM [58]. The integrin resides in focal adhesions which mediate this interaction; hence, focal adhesion binds the cytoskeletal protein with ECM. This interaction regulates the proliferation, migration, and differentiation of MSCs. Due to the differential regulation of ECM, integrin and cytoskeletal related genes, we predict the analogous interactions of ECM, integrin and cytoskeletal proteins would drive the MSC differentiation into NPCs.

Both GSEA and GO terms enrichment analyses showed that upregulated genes related to cell cycle-regulation in MSCs. Cell cycle regulation is crucial for enhanced MSC differentiation into adipocytes. One study reported that cell cycle arrest in G1 enhancing the differentiation of MSCs into adipocytes [59]. Microarray analysis also confirmed that during adipogenic differentiation from MSCs, cell cycle arrest genes were upregulated [60]. One other study reported that berberine enhanced the neural differentiation from neuroblastoma cells by cell cycle arrest [61]. In other study, the inhibition of cyclin-dependent kinase 4 (CDK4) led to the hypo phosphorylation of Smads and STAT3, which enhanced human MSC differentiation into NPCs [62]. In pluripotent stem cells such as embryonic stem cells, cell cycle regulation is also crucial for renewal and differentiation. The embryonic stem cells have shorter G1 and G2 compared with adult stem cells, but the G1 phase enlarges when embryonic stem cells are differentiated [63]. Differential regulation of cell cycle related genes indicate the cell cycle regulation may play a crucial role in the differentiation.

The other main aim of the current study was to compare the transcription factors between NPCs and MSCs to determine the transcription factors that are involved in the differentiation of MSCs into NPCs. Specific transcription factors are crucial in differentiation and trans-differentiation. These specific factors, particularly master transcription factors, bind to enhancers or upstream sequencers and regulate many genes, resulting in the differentiation or trans-differentiation of the cell [64]**.** Apart from a few transcription factors, such as MyoD, which enhance muscle differentiation, most transcription factors interact with others, forming a transcription factor network which maintains the phenotype of cells [65]. Fibroblast was induced into a pluripotent state through the ectopic expression of OCT4, SOX2, c-MYC, and KLIF4 in Fibroblasts [66]. These factors form and maintain pluripotency by forming a transcription factor network in embryonic stem cells [67].

MSCs can be derived from several tissues, and discovering core transcriptomes such as transcription factors that maintain MSC multipotency is crucial. In 2014, the transcriptomics of human MSCs from the bone marrow and the placenta were compared [68]. There was common and dissimilar genes expression between the two stem cell types. In another detailed study in 2016, the transcriptomes of human MSCs from the bone marrow, adipose tissues, placenta, and fibroblast cells were compared [69]. This meta-analysis revealed that human MSCs had 13 transcription factors that defined MSC multipotency: *ARID5B, CREB3, EPAS1, FHL2, GTF2E2, GTF2IRD1, ID3, LMO7, SNAI2, TAF13, TEAD3, TULP3,* and *ZNF532*. Since these transcription factors define the core of the MSCs lineage, their expression was assessed in the NPCs. *Creb3, Fhl2, Id3, Snai2,* and *Taf13* were downregulated in NPCs, confirming the MSCs were differentiated into NPCs.

Among the 104 transcription factors, the most highly significantly expressed transcription factor in NPCs was *Hes3*. *Hes3* is crucial for nervous development; inactivation of this gene leads to depletion of neural stem cells [70]. The second most significant transcription factor was *Tbx10*, which is a T-box binding protein that has a crucial role in motor neuron development [71]. Another significant transcription factor involved in nervous system development is *Nr4a1* [72]. The 104 upregulated transcription factors in NPCs have their role in neurogenesis. However, future studies should focus on finding the transcription factor network related to these 104 genes that define NPCs.

Transcription factors enrichment analysis was also performed by ChEA3 to find transcription factors that are associated with differential gene changes and they play roles in NPCs differentiation. The list of 3252 differentially expressed genes were subjected to transcription factor enrichment analysis [38]. Among the top ten enriched transcription factors (Fig. 7), the analysis revealed *Meox1* and *Aebp1* are significantly upregulated and *Osr1, Foxs1* and *Cenpa* are significantly downregulated in NPC. Foxs1 is a sensory neuron‐specific gene [73]. Interestingly, *Heyl*, a transcription factor known to promote neuronal differentiation was also identified among the top 3 transcription factors [74]. The complete list of all 1632 site-specific TFs covered by ChEA3, prioritized based on their integrated MeanRank score, along with the overlapping genes found to be differentially expressed by for each TF entry are listed in Additional file 3.

The vast transcriptomic expression difference between MSCs and NPCs indicate that these two cell types are distinct and unique transcriptomic profiles define the two cell types. Some of those differentially expressed genes, particularly the transcription factors can be used as markers of differentiation as well as they can be used further to optimise the differentiation of MSCs into NPCs.

## Conclusions

Overall, this study indicated that rat MSCs could be differentiated into NPCs after Day 3 of induction. NPCs have the capacity for terminal differentiation into neurons. The transcriptomic analysis identified a complex regulation of genes during early neural differentiation of rat MSCs into NPCs in the presence of EGF and bFGF. The transcriptomic data obtained from this study may provide valuable information regarding the biochemical process instigating neural differentiation of MSCs. The transcriptomic analysis revealed the separate gene expression profiles that define MSCs and NPCs.

The vast differences in the transcription factor expression profiles (particularly the transcription factor profiles) between NPCs and MSCs warrant further investigation. Future studies should seek to discover and describe the transcription factor network that defines and maintains NPCs.

## Supplementary information

**Additional file 1: Figure S1.** Representative flow cytometry analysis of Sox2 expression by MSC-derived NPCs and MSCs in basal media. **Figure S2.** Representative flow cytometry analysis of Beta-3-tubulin expression of terminally differentiated NPCs into neurons.

**Additional file 2.** Read pre-processing, quality control, alignment, and gene quantification. Before differentiation expression analysis, raw reads were evaluated for quality check in terms of sequencing quality and potential contamination. Trimming was performed using BBDuk. Reads were aligned to the latest reference genome (rn6) with GTF from Ensembl (v99) using STAR aligner. Aligned reads were transformed into read count per gene using RSEM tool.

**Additional file 3.** Detail of all differentially regulated genes and enriched gene ontology ontologies during the differentiation. Significantly enriched GO terms generated from ClueGO plugin for both up and downregulated genes in NPCs. Details of transcription factor enrichment analysis using ChEA3.

**Additional file 4.** Detail of dataset validation using quantitative real-time PCR. The expression of selected genes of interest was validated using quantitative real-time PCR.

**Additional file 5.** Full list of differential regulated transcription factors in MSCs-derived NPC.

## Data Availability

All data generated and/or analysed during this study are included in this published article or deposited in the GEO database NCBI (https://www.ncbi.nlm.nih.gov/geo/), with the accession number being GSE104548 (Temporary reviewer’s token: apkhkswkthmznqt). Any additional data used and analysed during the current study are available from the corresponding author on reasonable request.

## Abbreviations

MSCs: Mesenchymal stem cells
NPCs: Neural progenitor-like cells
bFGF: Basic fibroblast growth factor
EGF: Epidermal growth factor
GO: Gene ontology
iPSCs: Induced pluripotent stem cells
PCA: Principal component analysis
RT-qPCR: Quantitative real-time PCR
GSEA: Gene set enrichment analysis
ECM: Extracellular matrix
SD: Sprague Dawley
