# Supplementary material for: Significant transcriptomic changes are associated with differentiation of bone marrow-derived mesenchymal stem cells into neural progenitor-like cells in the presence of bFGF and EGF
Source: Cell Biosci. 2020 Oct 28;10:126. doi: 10.1186/s13578-020-00487-z (PMC7594431; doi:10.1186/s13578-020-00487-z)
Supplement: Supplementary file 1 — Additional file 1: Figure S1. Representative flow cytometry analysis of Sox2 expression by MSC-derived NPCs and MSCs in basal media. Figure S2. Representative flow cytometry analysis of Beta-3-tubulin expression of terminally differentiated NPCs into neurons. [file 13578_2020_487_MOESM1_ESM.pdf]

Figure S1

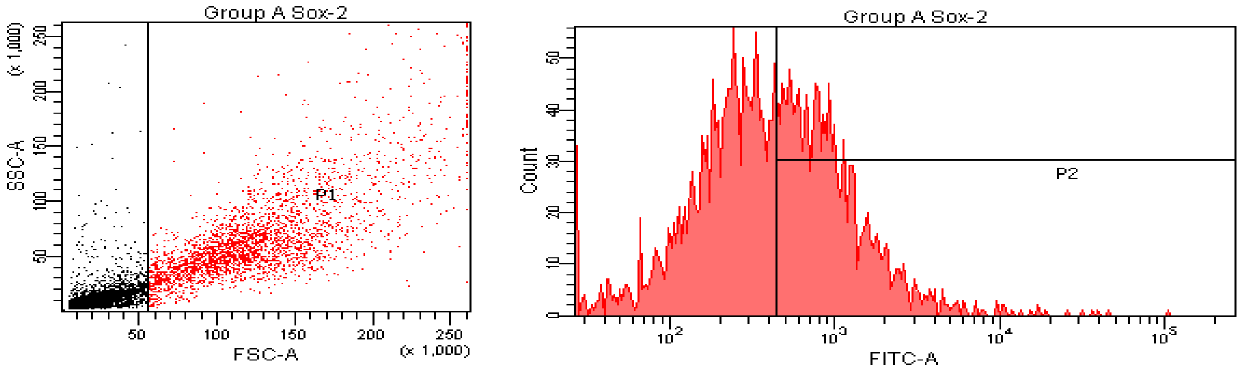

Tube: Group A Sox-2

Population	#Events	%Parent	%Total
All Events	10,000	####	100.0
P1	3,051	30.5	30.5
P2	1,453	47.6	14.5

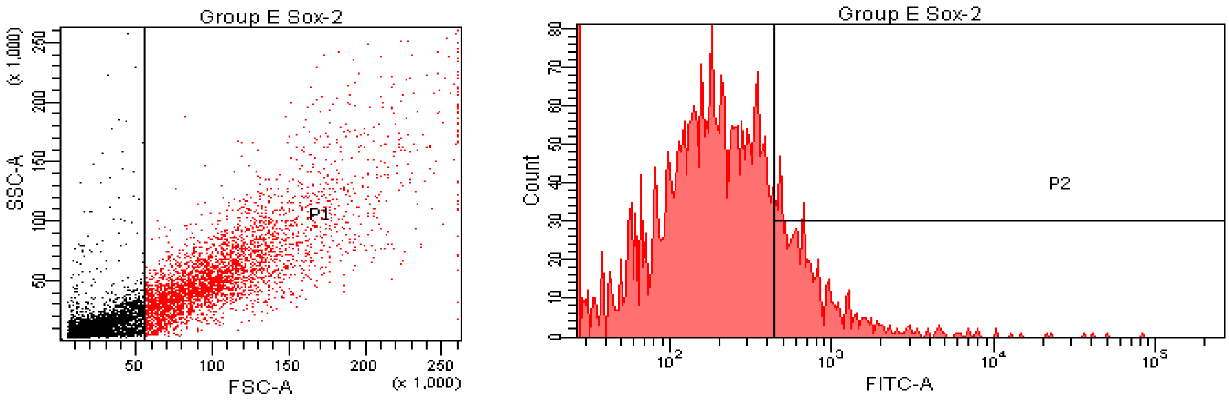

Tube: Group E Sox-2

Population	#Events	%Parent	%Total
All Events	10,000	####	100.0
P1	3,674	36.7	36.7
P2	680	18.5	6.8

Figure S1: The expression of Sox2 expression in the MSC-derived NPCs (Group A) and the control where the MSCs were suspended in NeuroCult® NS-A proliferation media without EGF and FGF (Group E).

Figure S2

A:

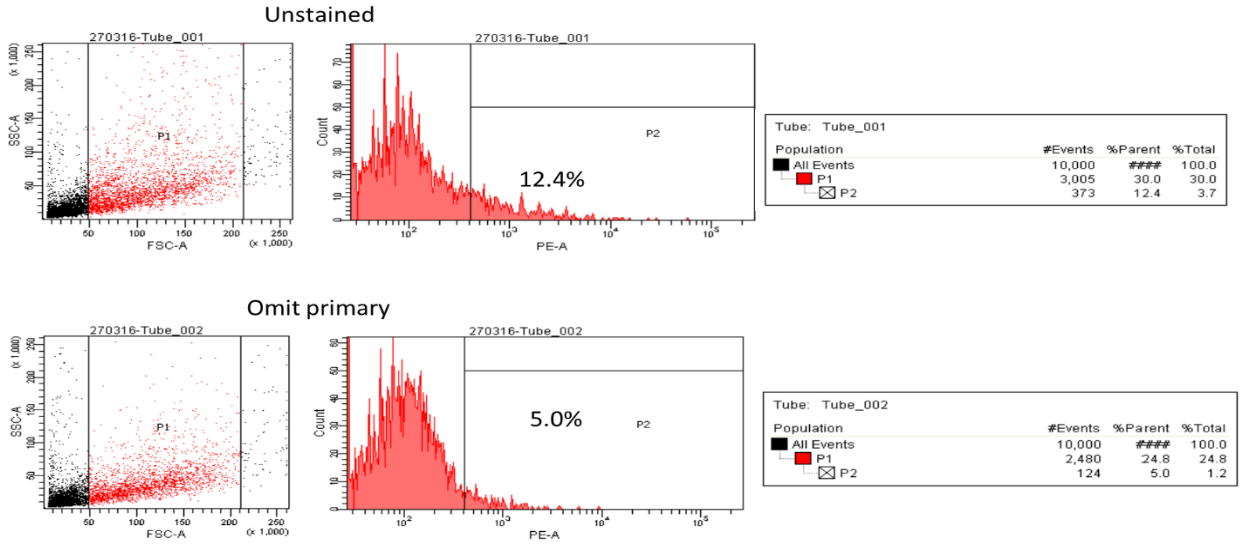

B

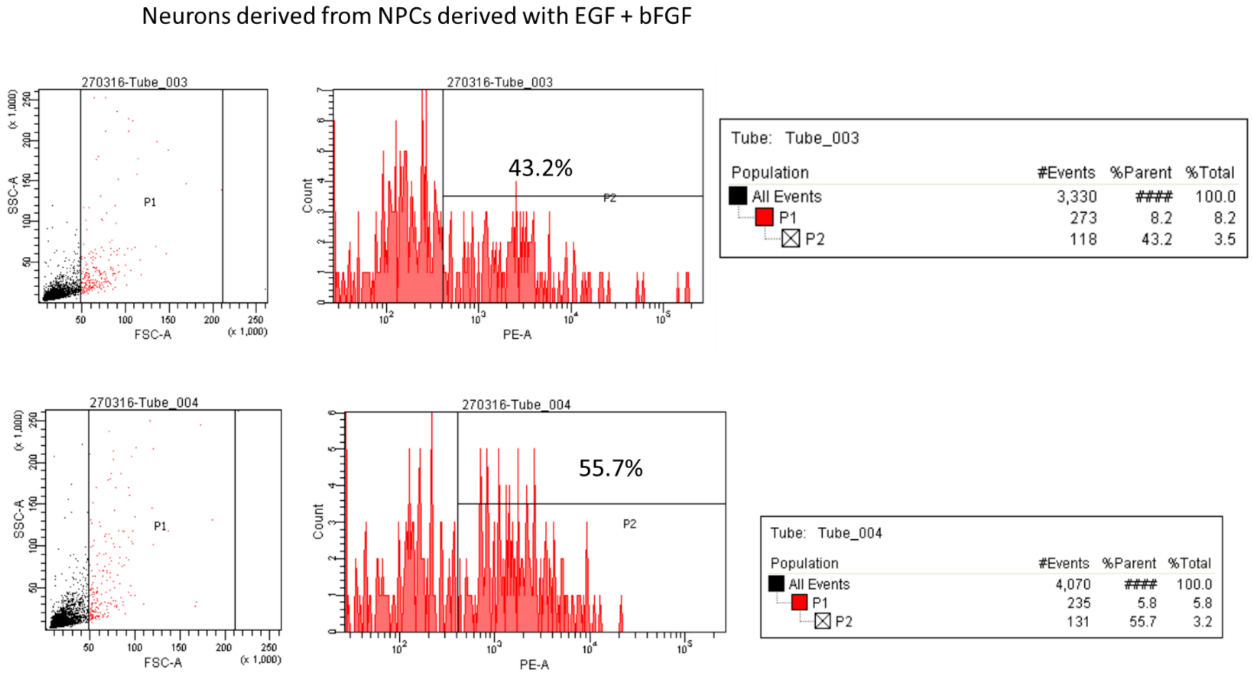

Figure S2: The above figure shows the expression of Beta Tubulin III for mature neurons. A: expression of the Beta Tubulin III in unstained and the negative control. B: expression of Beta Tubulin III in the derived neurons from NPCs
