# Supplementary material for: Significant transcriptomic changes are associated with differentiation of bone marrow-derived mesenchymal stem cells into neural progenitor-like cells in the presence of bFGF and EGF
Source: Cell Biosci. 2020 Oct 28;10:126. doi: 10.1186/s13578-020-00487-z (PMC7594431; doi:10.1186/s13578-020-00487-z)
Supplement: Supplementary file 2 — Additional file 2. Read pre-processing, quality control, alignment, and gene quantification. Before differentiation expression analysis, raw reads were evaluated for quality check in terms of sequencing quality and potential contamination. Trimming was performed using BBDuk. Reads were aligned to the latest reference genome (rn6) with GTF from Ensembl (v99) using STAR aligner. Aligned reads were transformed into read count per gene using RSEM tool. [file 13578_2020_487_MOESM2_ESM.pdf]

QC & MAPPING STATISTICS

The following sections provide a summary of the quality controls obtained for your dataset.

QC summary

This section includes QC and statistics after pre processing of the reads – trimming for adapters and low quality bases and removal of rRNA reads.

On an average 96% of the reads corresponding to 51.86 M reads pass the filtering criteria across all the samples.

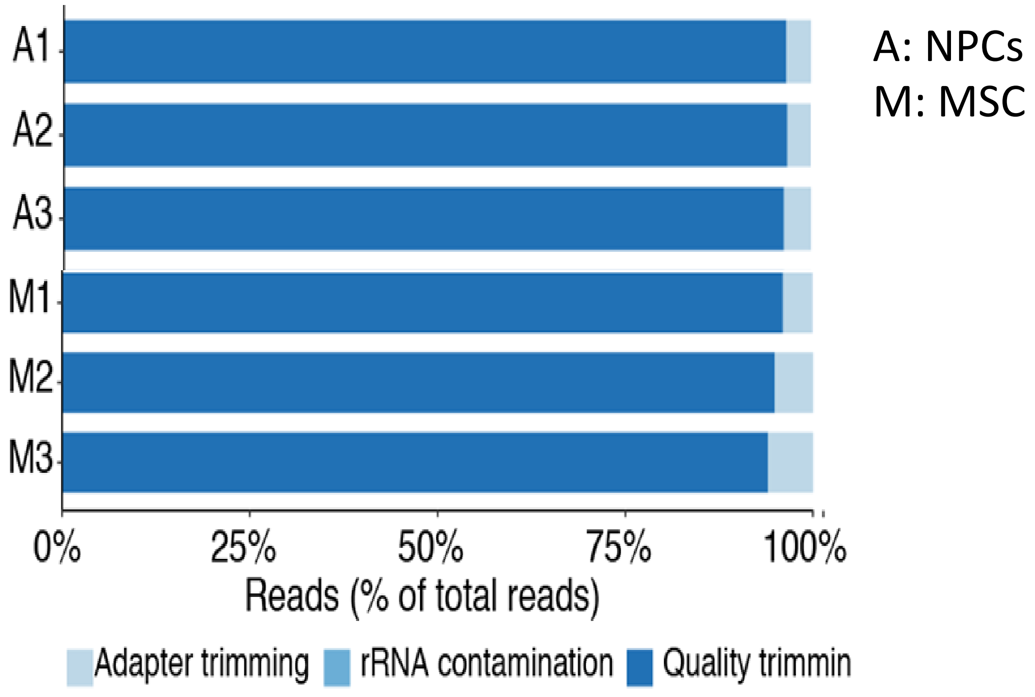

QC statistics plot of samples

Sample	Raw reads	Adapter trimming	rRNA contamination	Quality trimming
**A1**	49.05	49.04	47.44	47.44
**A2**	47.41	47.4	45.9	45.9
**A3**	60.28	60.27	58.09	58.09
**M1**	52.15	52.12	50.04	50.04
**M2**	62.01	62.01	58.83	58.83
**M3**	52.85	52.84	49.66	49.66

QC statistics of samples. *Numbers are represented in millions*

Mapping Summary

This section includes the mapping statistics on the rat genome by STAR alignment tool.
On an average 97% of the reads corresponding to 50.5 M reads mapped on the genome across all the samples. (Figure 2 & Table 2)

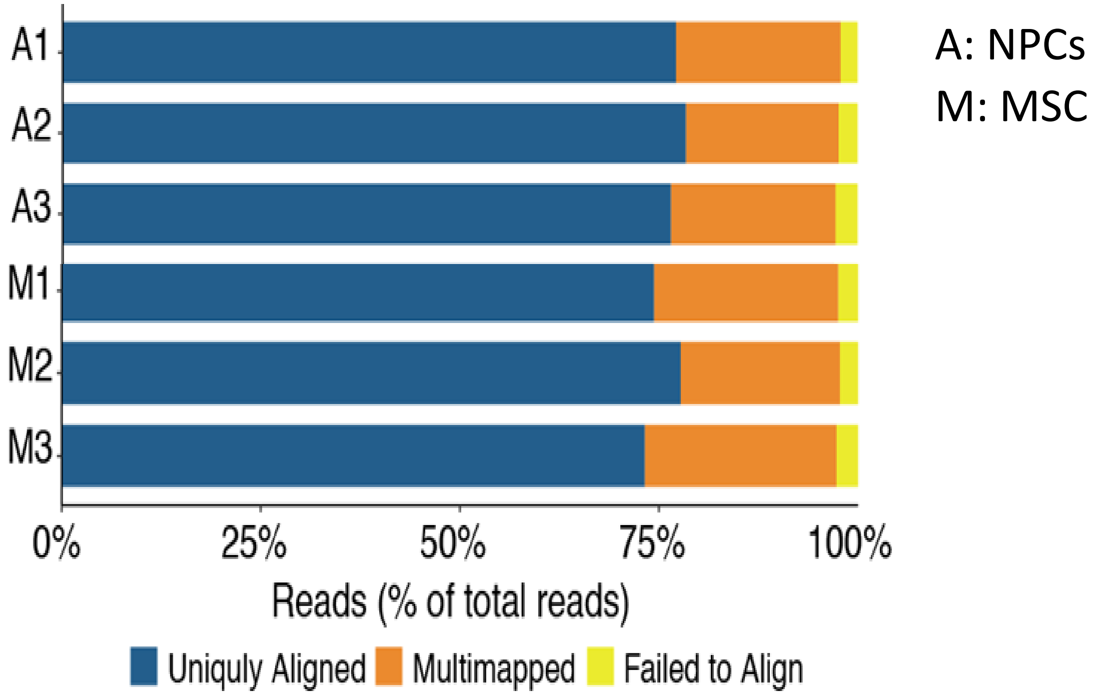

Mapping statistics plot of samples

Sample	# Reads	Uniquely Aligned	Multi-mapped	Aligned	Failed to Align
**A1**	47.44	36.62	9.82	46.44	1
**A2**	45.9	35.98	8.82	44.8	1.1
**A3**	58.09	44.46	12.01	56.47	1.62
**M1**	50.04	37.22	11.58	48.79	1.25
**M2**	58.83	45.74	11.76	57.5	1.33
**M3**	49.66	36.37	11.96	48.32	1.34

Mapping statistics of samples. *Numbers are represented in millions*
